# A new nomogram for assessing complete response (CR) in gastric diffuse large B-cell lymphoma (DLBCL) patients after chemotherapy

**DOI:** 10.1007/s00432-023-04862-4

**Published:** 2023-05-29

**Authors:** Ping Wang, Kaige Chen, Jiayang Wang, Zihao Ni, Naijian Shang, Wei Meng

**Affiliations:** 1 Radiology Department, Harbin Medical University, Harbin Medical University Cancer Hospital, 150 Haping Road, Harbin, 150081 Heilongjiang China; 2 Department of Ultrasound, Harbin Medical University, Harbin Medical University Cancer Hospital, 150 Haping Road, Harbin, 150081 Heilongjiang China

**Keywords:** Gastric diffuse large B-cell lymphoma, Chemotherapy, Complete response, Radiography

## Abstract

**Purpose:**

Achieving complete response (CR) after first-line chemotherapy in gastric DLBCL patients often results in longer disease-free survival. We explored whether a model based on imaging features combined with clinicopathological factors could assess the CR to chemotherapy in patients with gastric DLBCL.

**Methods:**

Univariate (*P* < 0.10) and multivariate (*P* < 0.05) analyses were used to identify factors associated with a CR to treatment. As a result, a system was developed to evaluate whether gastric DLBCL patients had a CR to chemotherapy. Evidence was found to support the model's ability to predict outcomes and demonstrate clinical value.

**Results:**

We retrospectively analysed 108 people who had been diagnosed gastric DLBCL; 53 were in CR. Patients were divided at random into a 5:4 training/testing dataset split. β2 microglobulin before and after chemotherapy and lesion length after chemotherapy were independent predictors of the CR of gastric DLBCL patients after chemotherapy. These factors were used in the predictive model construction. In the training dataset, the area under the curve (AUC) of the model was 0.929, the specificity was 0.806, and the sensitivity was 0.862. In the testing dataset, the model had an AUC of 0.957, specificity of 0.792, and sensitivity of 0.958. The AUC did not differ significantly between the training and testing dates (*P* > 0.05).

**Conclusion:**

A model constructed using imaging features combined with clinicopathological factors could effectively evaluate the CR to chemotherapy in gastric DLBCL patients. The predictive model can facilitate the monitoring of patients and be used to adjust individualised treatment plans.

## Introduction

DLBCL is the most prevalent kind of non-Hodgkin lymphoma (NHL), which is a category of diseases with high heterogeneity. DLBCL accounts for approximately 30–40% of NHL cases (Vaidya and Witzig 2014). About a quarter of all instances of DLBCL are diagnosed at an early stage, known as limited-stage lymphoma, and are classified as stage I or II. 75% of DLBCL cases are diagnosed at more advanced stages (stages III and IV) (Cottereau et al. 2021). Chemotherapy is the main treatment modality for NHL, and the standard regimen is a combination of rituximab and anthracycline, such as R-CHOP (rituximab, cyclophosphamide, hydroxydaunorubicin hydrochloride, Oncovin® [vincristine], and prednisone) (Maurer et al. 2016; Hu et al. 2019). However, the treatment response varies widely, ranging from no tumour regression to complete response (CR). The 5-year survival rate associated with R-CHOP treatment is approximately 50–55%. In addition, 40–50% of patients develop relapsed or refractory DLBCL (Fan et al. 2021). Achieving complete response after the first course of induction chemotherapy is critical, as it often results in long-term progression-free survival (Zhang et al. 2015; Hawkes et al. 2018).

Imaging plays an important role in assessing the response to treatment in patients with gastric DLBCL (Kwee et al. 2008). In a previous study, follow-up was performed according to the National Comprehensive Cancer Network guidelines in which computed tomography (CT) is recommended every 6 months for 2 years after treatment completion. However, many patients underwent more scans than recommended by the National Comprehensive Cancer Network (Abel et al. 2012). Studies have also suggested that positron emission tomography (PET) should be performed 6–8 weeks after chemoimmunotherapy (Zelenetz et al. 2013). With growing evidence supporting the central role of PET–CT in NHL staging and response assessment (Gómez León et al. 2017; Wu et al. 2014), a five-point scale has been developed to visually assess varying degrees of response in the middle and at the end of treatment; however, there was a wide range of subjective differences in people used the five-point scale to assess different levels of response at the middle and end of treatment (Thompson et al. 2014). Therefore, visual assessment is still challenging, and a method is urgently needed to help clinicians accurately assess the post-treatment response of patients with gastric DLBCL.

To date, no study has evaluated the CR after treatment in gastric DLBCL patients using a predictive model. Therefore, the goals of this research were to conduct a screening for characteristics associated with CR after treatment in gastric DLBCL patients, develop a predictive model, and create a nomogram, and verify the accuracy and clinical validity of the model.

## Materials and methods

### Patients and reference standard

We retrospectively analysed all patients with pathologically confirmed gastric DLBCL between January 2017 and October 2022. Finally, 108 patients (aged 25–78 years; mean age: 52.5 years) were included. The inclusion criteria were as follows: (1) histopathologically diagnosed gastric DLBCL for the first time; (2) complete clinical and pathological information and auxiliary examination results; and (3) acceptance of a complete treatment cycle in our hospital. The exclusion criteria were (1) poor image quality owing to various reasons and (2) local or systemic antitumour therapy before chemotherapy.

We used a random 5:4 split to separate the patients into our training and testing datasets. The model was developed using the training dataset, and its accuracy was checked using the testing dataset. Our institution's ethics committee green-lighted this retrospective study without requiring participants to give their permission.

### Treatment and follow-up

**Fig. 1:**
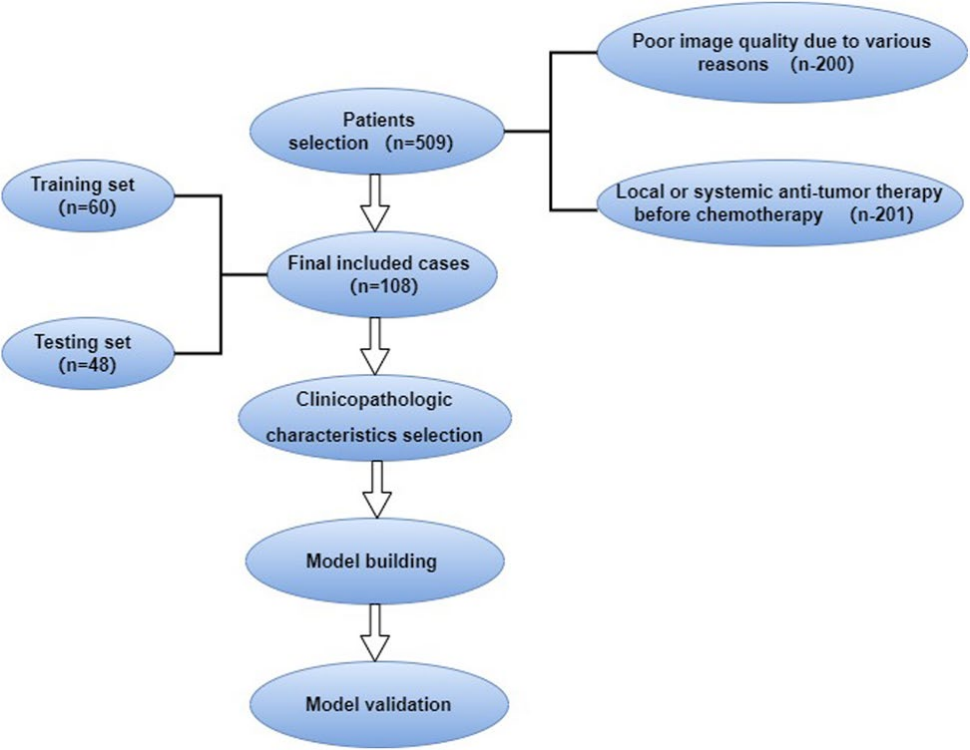
Study workflow. *n* number of patients

All patients with gastric DLBCL received standard treatment within 1 week after CT, and the treatment plan was six cycles of R-CHOP alone. The response to treatment was determined based on post-treatment physical condition and information gathered from the PET–CT scan results. The patients' electronic medical record (EMR) was mined for information on their clinical and pathological details (Table 1). Routine blood tests for lactate dehydrogenase (LDH) and β2 microglobulin were performed within 1 week before chemotherapy and within 1 week of the first cycle of chemotherapy. Normal levels of LDH and β2 microglobulin at our hospital are 120–246 U/L and 0–3.0 mg/L, respectively. Before treatment, the lesion length and longest diameter of the largest lymph node were simultaneously measured by two radiologists on the venous phase images, and the average value of the two measurements was calculated. In addition, the longest diameter of the lesion after treatment and the longest diameter of the largest lymph node were quantified on the PET–CT scans simultaneously by two radiologists, and the average of the two measurements was calculated. The Ann Arbor Staging System was used to stage all of the included patients (Carbone et al. 1971) using the first contrast-enhanced CT examination. The study process is shown in Fig. 1.

**Table 1:** Analysis of clinical characteristics of CR and non-CR

Characteristics	CR *n* = 53	non-CR *n* = 55	*P*
Gender	–		0.054
Female	31 (58.5%)	21 (38.2%)	–
Male	22 (41.5%)	34 (61.8%)	–
Pre-β_2_-microglobulin (mg/L)	1.94 (0.77)	2.49 (1.02)	0.002
Post-β_2_-microglobulin (mg/L)	1.46 (0.6)	2.36 (1.1)	0.001
Pre-LDH (U/L)	190 (71)	189 (100)	0.480
Post-LDH (U/L)	185 (63)	217 (91)	0.002
Pre-length of lesion (mm)	51 (63.4)	73.93 (40.9)	0.005
Post-length of lesion (mm)	7 (4.7)	32 (54.6)	0.002
Pre-long diameter of lymph node (mm)	7 (6)	20 (36)	0.003
Post-long diameter of lymph node (mm)	3 (2)	8 (8)	0.001
Location	–	–	0.104
Gastric fundus	4 (7.5%)	0 (0%)	–
Gastric body	19 (35.8%)	22 (40.0%)	–
Gastric antrum	21 (39.6%)	22 (40.0%)	–
Gastric horn	7 (13.2%)	8 (14.5%)	–
Large part of stomach	0 (0%)	3 (5.5%)	–
Cardia	2 (3.8%)	0 (0%)	–
Ann Arbor staging	–	–	0.002
I	30 (56.6%)	12 (21.8%)	–
II	21 (39.6%)	31 (56.4%)	–
III	1 (1.9%)	3 (5.5%)	–
IV	1 (1.9%)	9 (16.4%)	–

The *N* in parentheses, the number of people

CR complete response, non-CR non-complete response

*P* value, the significance of differences between the characteristics in complete response and non-complete response

*P* < 0.05, statistical significance

### Clinical feature selection

Clinical variables relevant to the CR after treatment in gastric DLBCL patients include sex, pre-chemotherapy β2 microglobulin, post-chemotherapy β2 microglobulin, pre-chemotherapy LDH, post-chemotherapy LDH, pre-chemotherapy lesion length, post-chemotherapy lesion length, pre-chemotherapy longest lymph-node diameter, post-chemotherapy longest lymph-node diameter, tumour location, and stage. We first used univariate logistic regression analysis to identify candidate clinical factors related to gastric DLBCL response after treatment (*P* < 0.10) and then used multivariate logistic regression analysis to determine the independent predictors related to CR after treatment in gastric DLBCL patients (*P* < 0.05) for model development.

### Model building and verification

We developed a nomogram (Kattan and Marasco 2010) including clinical features to provide clinicians with a quantitative tool to assess the CR of gastric DLBCL patients after chemotherapy. A testing dataset was used to validate the model's performance. Receiver-operating characteristic (ROC) curves were used to measure the ability of the model to evaluate the response after gastric DLBCL treatment. The nomogram's clinical usefulness was evaluated with the help of decision curve analysis [17], which quantified the net benefit at varying levels of certainty. Calibration curves (Li et al. 2021) were used to reflect the agreement between the predicted model and the observations.

### Statistical analyses

For this retrospective study, we employed the Kruskal–Wallis test for continuous and ordinal variables and either the chi-squared or Fisher's exact test for categorical data. The model's discrimination, sensitivity, and specificity for the occurrence of events were measured using the ROC and AUC curves. A significance level of *P* < 0.050 was used. The nomogram and model assessment were carried out in R language (version 4.1.1; R Foundation for Statistical Computing, Vienna, Austria). The data were analysed statistically by use of SPSS (version 17.0; SPSS Inc., Chicago, IL). Statspackage (version 4.1.1) in R software was used for single and multi-factor logistic regression analysis. pROCpackage (version 1.18.0) was used for ROC curve. ModelGood (version 1.0.9) was used to draw calibration curve. Plot decision curves were plotted using ggDCA (version 1.2).

## Results

### Clinical and pathological characteristics

The clinical and pathological features of the 108 patients are shown in Table 1. CR was observed in 53 patients (49.07%). There was no significant difference in sex, LDH level before chemotherapy, or tumour location between the CR and non-CR groups (*P* > 0.05). However, β2 microglobulin before and after chemotherapy, LDH after chemotherapy, lesion length before and after chemotherapy, longest diameter of the lymph node before and after chemotherapy, and stage differed significantly between the two groups (*P* < 0.05). There were no significant differences (*P* < 0.05) in clinical and pathological features between the testing and training datasets (Table 2).

**Table 2:** Analysis of clinical characteristics of training dataset and testing dataset

Characteristics	Training dataset *n* = 60	Testing dataset *n* = 48	*P*
Gender	–	–	0.966
Female	29 (48.3%)	23 (47.9%)	–
Male	31 (51.7%)	25 (52.1%)	–
Pre-β_2_-microglobulin (mg/L)	2.215 (1.03)	2.14 (0.92)	0.346
Post-β_2_-microglobulin (mg/L)	1.93 (1.1)	1.82 (1.3)	0.441
Pre-LDH (U/L)	192.5 (92)	177 (51)	0.178
Post-LDH (U/L)	203.5 (71)	187 (85)	0.339
Pre-length of lesion (mm)	70 (51)	68.05 (70.6)	0.951
Post-length of lesion (mm)	12 (31.3)	11.95 (27.8)	0.788
Pre-long diameter of lymph node (mm)	10 (22)	10 (19)	0.887
Post-long diameter of lymph node (mm)	4.6 (4)	4.85 (7)	0.916
Location	–	–	0.324
Gastric fundus	1 (1.7%)	3 (6.3%)	–
Gastric body	20 (33.3%)	21 (43.8%)	–
Gastric antrum	29 (48.3%)	14 (29.2%)	–
Gastric horn	8 (13.3%)	7 (14.6%)	–
Large part of stomach	1 (1.7%)	2 (4.2%)	–
Cardia	1 (1.7%)	1 (2.1%)	–
Ann Arbor staging	–	–	0.854
I	22 (36.7%)	20 (41.7%)	–
II	31 (51.7%)	21 (43.8%)	–
III	2 (3.3%)	2 (4.2%)	–
IV	5 (8.3%)	5 (10.4%)	–

The *N* in parentheses, the number of people

*P* value, the significance of differences between the training and testing dataset

*P* < 0.05, statistical significance

### Clinical feature selection

Univariate analysis (*P* < 0.10) showed that β2 microglobulin before and after chemotherapy, LDH after chemotherapy, lesion length after chemotherapy, longest diameter of the lymph node before and after chemotherapy, and stage were significantly associated with treatment response after chemotherapy in patients with gastric DLBCL. All seven significant variables were included in the multivariate analysis, and β2 microglobulin before chemotherapy and lesion length after chemotherapy were statistically significant (Table 3). Although β2 microglobulin levels after chemotherapy were not statistically significant after multivariate logistic regression analysis, according to the literature (Wang et al. 2015), we believe that β2 microglobulin after chemotherapy is an independent predictor of the CR after treatment in gastric DLBCL patients. Therefore, β2 microglobulin before chemotherapy, β2 microglobulin after chemotherapy, and lesion length after chemotherapy were selected as independent predictors of the CR after treatment in gastric DLBCL patients.

**Table 3:** Analysis of clinicopathological risk factors associated with CR

Characteristics	Univariate logistic regression			Multivariate logistic regression		
	OR	95% CI	P*	OR	95% CI	P**
Gender	0.446	0.159–1.253	0.126	–	–	–
Female	–	–	–	–	–	–
Male	–	–	–	–	–	–
Pre-β_2_-microglobulin (mg/L)	0.255	0.098–0.665	0.005	742.525	1.436–384,010.791	0.038
Post-β_2_-microglobulin (mg/L)	0.090	0.025–0.324	0.001	0.000	0.000–1.788	0.063
Pre-LDH (U/L)	0.997	0.991–1.003	0.360	-	-	-
Post-LDH (U/L)	0.979	0.965–0.994	0.007	0.881	0.763–1.017	0.083
Pre-length of lesion (mm)	0.991	0.976–1.006	0.236	–	–	–
Post-length of lesion (mm)	0.914	0.866–0.964	0.001	0.844	0.716–0.994	0.042
Pre-long diameter of lymph node (mm)	0.905	0.849–0.965	0.002	0.827	0.664–1.030	0.090
Post-long diameter of lymph node(mm)	0.579	0.415–0.809	0.001	0.218	0.034–1.388	0.107
Location	0.780	0.423–1.438	0.426	–	–	–
Gastric fundus	–	–	–	–	–	–
Gastric body	–	–	–	–	–	–
Gastric antrum	–	–	–	–	–	–
Gastric horn	–	–	–	–	–	–
Large part of stomach	–	–	–	–	–	–
Cardia	–	–	–	–	–	–
Ann Arbor staging	0.395	0.180–0.866	0.020	0.140	0.003–5.786	0.301
I	–	–	–	–	–	–
II	–	–	–	–	–	–
III	–	–	–	–	–	–
IV	–	–	–	–	–	–

CR complete response

*P < 0.1, statistical significance

**P < 0.05, statistical significance

### The new nomogram

**Fig. 2:**
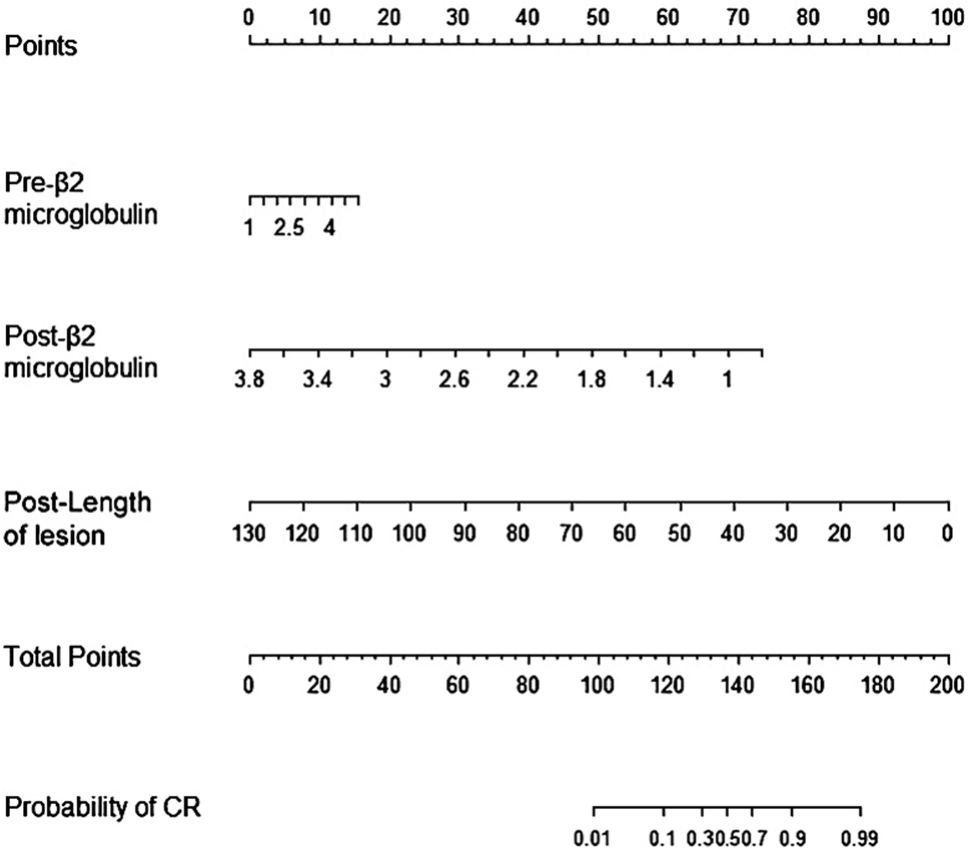
Nomogram

**Fig. 3:**
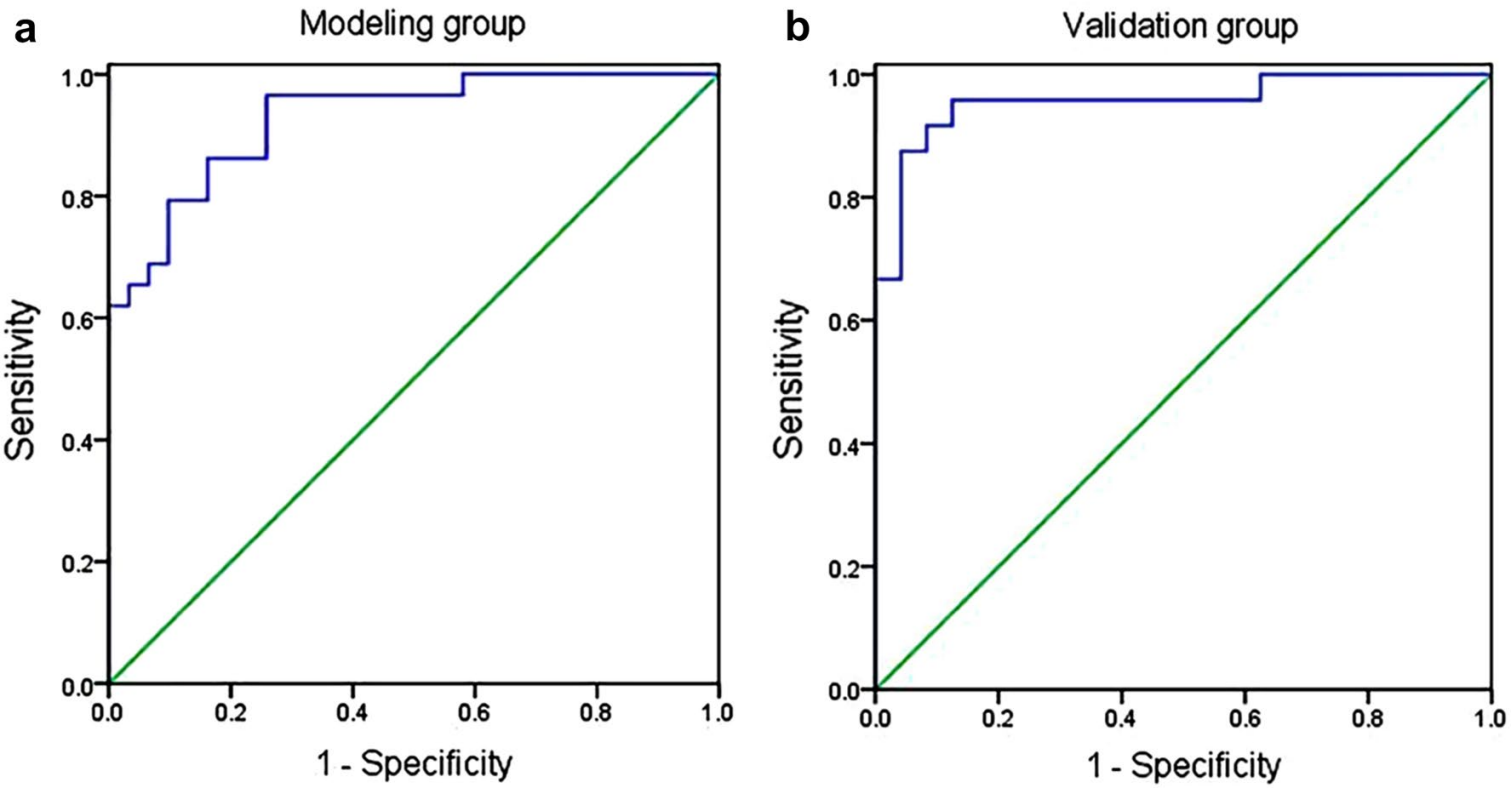
ROC curves, ROC, receiver-operating characteristic. ROC for the training dataset (**a**) and testing dataset (**b**)

**Fig. 4:**
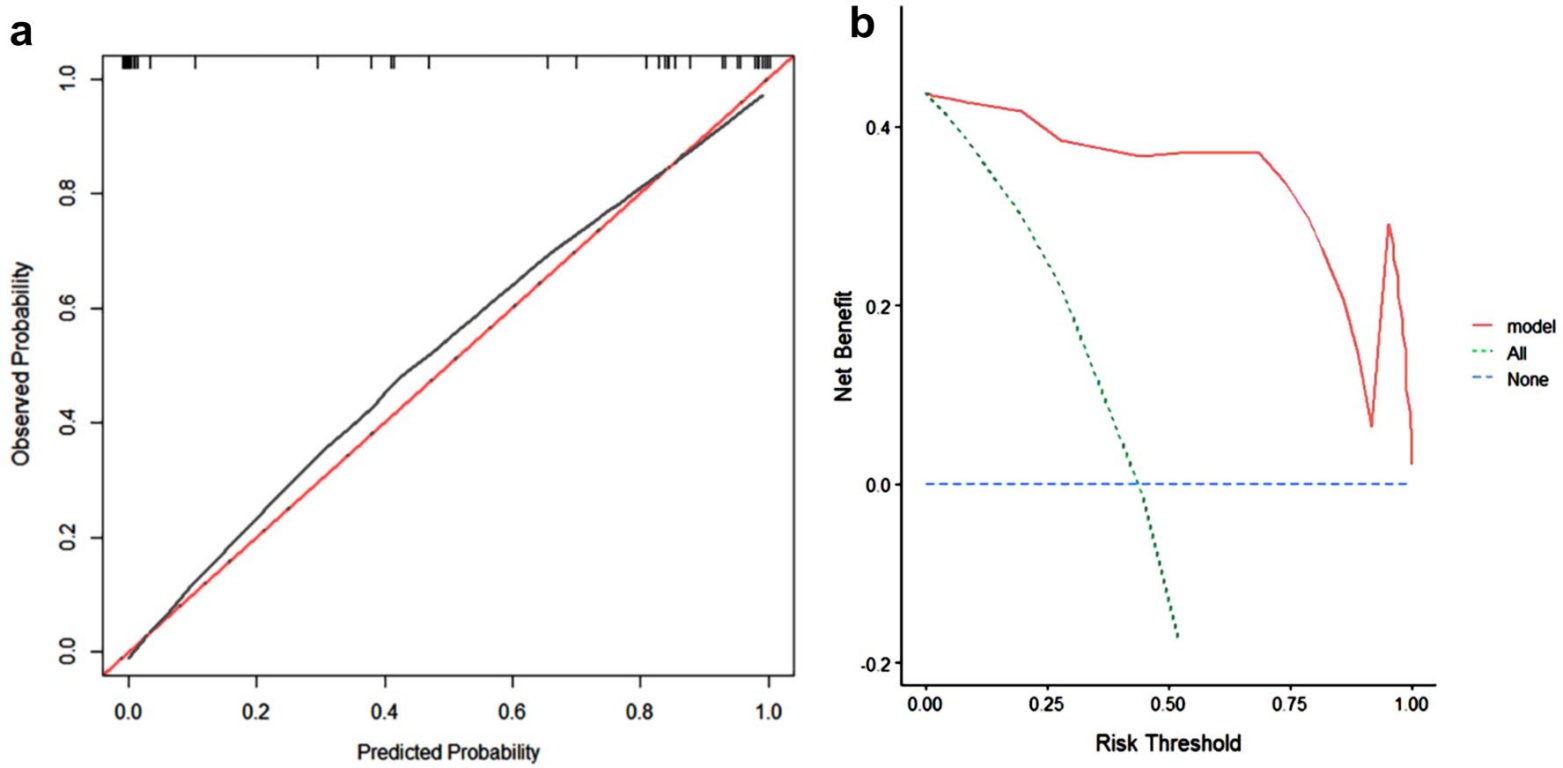
Calibration curves and DCA curves. Calibration curves for the testing dataset (**a**). DCA for the testing dataset (**b**). DCA decision curve analysis

Based on these three independent prognostic factors, a model and nomogram for evaluating the CR after treatment in gastric DLBCL patients were constructed (Fig. 2). The probability of each predicted value can be converted into a score based on the scale at the top of the chart. The corresponding predicted probabilities were then summed. The bottom of the series shows the model-assessed probability of CR after chemotherapy in patients with gastric DLBCL, and the ROC curve (Fig. 3) shows the discriminative performance of the predictive model. In the training dataset, the model achieved an AUC of 0.929, a specificity of 0.806, and a sensitivity of 0.862 (Fig. 3a). In the testing dataset, the model had an AUC of 0.957, a specificity of 0.792, and a sensitivity of 0.958 (Fig. 3b). There was no statistically significant difference in the AUC between the training and testing groups (*P* > 0.05), indicating that the prediction model has a high discriminative ability. The calibration curve (Fig. 4a) showed good agreement between the predicted and actual probabilities in the testing dataset, indicating a high degree of calibration for the predictive model (*P* > 0.05). Decision curve analysis of the testing dataset (Fig. 4b) showed that the model had high clinical utility when the threshold probability was 0.033–0.879.

## Discussion

Following chemotherapy in gastric DLBCL, treatment responses vary widely, ranging from no tumour regression to complete remission (Yin et al. 2014). The standard of complete remission is based on the new consensus reached by the International Conference of the Imaging of Malignant Lymphoma Working Group published by the Journal of Clinical Oncology, which recommends the use of a visual judgement method to report the results of PET–CT using a five-point scale [(Deauville) standard], combined with the patients’ expected prognosis, clinical manifestations, and other response indicators for joint interpretation (Barrington et al. 2014). The assessment of CR after gastric DLBCL treatment largely depends on radiologists’ experience; therefore, the assessment of CR after treatment in patients with gastric DLBCL remains challenging. In this retrospective study, we screened for independent factors associated with CR after chemotherapy in patients with gastric DLBCL and developed and validated a model for individualised noninvasive assessment of CR. To the best of our knowledge, no study has been conducted on the evaluation of CR after gastric DLBCL treatment using nomograms. Our study has important implications for the treatment and prognosis of patients with gastric DLBCL as the nomogram could be used to monitor patients and personalise their treatment.

Univariate and multivariate analyses revealed that β2 microglobulin levels before chemotherapy and lesion length after chemotherapy were independent predictors of CR in patients with gastric DLBCL. Wilder et al. (2001) demonstrated that bulky disease was a poor prognostic factor in patients with aggressive lymphoma treated with cyclophosphamide, doxorubicin, vincristine, and prednisone chemotherapy, with or without radiation, which is consistent with our study findings. Fang et al. (2019) showed that an abnormally elevated β2 microglobulin level is an independent factor affecting the prognosis of patients with gastric DLBCL; however, in our study, the P value of β2 microglobulin after chemotherapy was more than 0.05, which may be related to the small number of patients included. Therefore, we included post-chemotherapy β2 microglobulin levels in the predictive model.

Kim et al. (2007) used absolute lymphocyte counts to predict the response of gastric DLBCL to chemotherapy; however, their study considered haematological features and did not include imaging features. Studies (El-Galaly et al. 2014; Yang et al. 2011) have confirmed that imaging examinations play an important role in the assessment of response in patients with gastric DLBCL after treatment. Yoon et al. (2016) explored the utility of endoscopy during and after DLBCL treatment. They suggested that in addition to radiological examinations, endoscopy and biopsy should also be performed; however, the number of patients included in their study was small, only 45 cases; thus, there may be selection bias. Moreover, endoscopy and biopsy are invasive examinations, which increases the financial burden on patients. Wu et al. (2013) examined several magnetic resonance imaging sequences for the detection of lesions and the assessment of treatment response in gastric DLBCL patients. The diagnostic precision and prognosis utility of magnetic resonance imaging may be enhanced by new complementary techniques, such as diffusion-weighted imaging (DWI) with apparent diffusion coefficient; however, DWI has some drawbacks as well. DWI is artefact-sensitive, and DWI based on echo-planar imaging is susceptible to image distortion. In addition, the study by Wu et al. included a small sample of only 18 patients with histologically confirmed gastric DLBCL and lacked independent validation. Trotman et al. (2011) proposed that PET–CT is more accurate in evaluating the end of treatment, but after treatment, an inflammatory reaction will occur around the lesion, which will increase the local uptake of contrast agent in the lesion and interfere with the doctors’ assessment of complete remission. The main focus of the current study was to build a model that integrates clinical features and indirect radiation features to quantitatively evaluate the CR of patients with gastric DLBCL after chemotherapy, eliminating the influence of subjective factors. In the training dataset, the AUC of the model was 0.929, specificity was 0.806, and sensitivity was 0.862. In the testing dataset, the AUC of the model was 0.957, specificity was 0.792, and sensitivity was 0.958. The model has good evaluation ability, and its advantages are as follows. First, our model is simple and convenient and includes quantitative features. Second, in the PET–CT images, we used the length of the lesion to represent its size, and two doctors reassessed the lesion length. Although this assessment method has measurement errors, including lesion length is necessary, because studies (Pfreundschuh et al. 2008) have shown that lesion size is one of the risk factors affecting the prognosis of patients with gastric DLBCL. Finally, we quantified the predictive model using a visualisation and interpretation tool, the nomogram, which is an easy-to-use personalised decision-making tool that helps clinicians accurately assess the response of patients with gastric DLBCL to chemotherapy and facilitates personalised precision medicine. Accordingly, we believe that the proposed model is reliable and stable and can help radiologists to make more accurate diagnoses.

This study had several limitations. First, a small number of quantitative features were extracted from the PET–CT images, and the model only considered the length of the lesion. Therefore, selection bias may have occurred in our analysis. Second, the number of patients we included is small, and most of them are concentrated in phase I and phase II. In future studies, we will further expand the number of patients; third, this was a single-centre study, and prospective multicentre experimental studies are needed to validate the model experimentally. In the future, we will consider using radiomics to evaluate the response of patients with gastric DLBCL after chemotherapy and explore more clinical risk factors related to the prognosis of these patients, such as lesion volume and metabolic tumour burden.

## Conclusion

A model constructed using indirect imaging features combined with clinicopathological factors could effectively evaluate the response of patients with gastric DLBCL after chemotherapy. Our findings are of great significance as the model could be used to facilitate careful and regular patient monitoring, accurately evaluate the treatment response, and adjust the individualised treatment plans.

## Data Availability

The data that support the findings of this study were available upon request from the corresponding author. The data were not publicly available due to privacy or ethical restrictions.
